# The Roles of Vitamin C in Skin Health

**DOI:** 10.3390/nu9080866

**Published:** 2017-08-12

**Authors:** Juliet M. Pullar, Anitra C. Carr, Margreet C. M. Vissers

**Affiliations:** Department of Pathology, University of Otago, Christchurch, P.O. Box 4345, Christchurch 8140, New Zealand; juliet.pullar@otago.ac.nz (J.M.P.); anitra.carr@otago.ac.nz (A.C.C.)

**Keywords:** ascorbate, dermis, epidermis, skin barrier function, vitamin C status, skin aging, wound healing, collagen, UV protection

## Abstract

The primary function of the skin is to act as a barrier against insults from the environment, and its unique structure reflects this. The skin is composed of two layers: the epidermal outer layer is highly cellular and provides the barrier function, and the inner dermal layer ensures strength and elasticity and gives nutritional support to the epidermis. Normal skin contains high concentrations of vitamin C, which supports important and well-known functions, stimulating collagen synthesis and assisting in antioxidant protection against UV-induced photodamage. This knowledge is often used as a rationale for the addition of vitamin C to topical applications, but the efficacy of such treatment, as opposed to optimising dietary vitamin C intake, is poorly understood. This review discusses the potential roles for vitamin C in skin health and summarises the in vitro and in vivo research to date. We compare the efficacy of nutritional intake of vitamin C versus topical application, identify the areas where lack of evidence limits our understanding of the potential benefits of vitamin C on skin health, and suggest which skin properties are most likely to benefit from improved nutritional vitamin C intake.

## 1. Introduction

The skin is a multi-functional organ, the largest in the body, and its appearance generally reflects the health and efficacy of its underlying structures. It has many functions, but its fundamental role is to provide a protective interface between the external environment and an individual’s tissues, providing shielding from mechanical and chemical threats, pathogens, ultraviolet radiation and even dehydration (functions reviewed in [1]). Being in constant contact with the external environment, the skin is subject to more insults than most of our other organs, and is where the first visible signs of aging occur.

**Figure 1:**
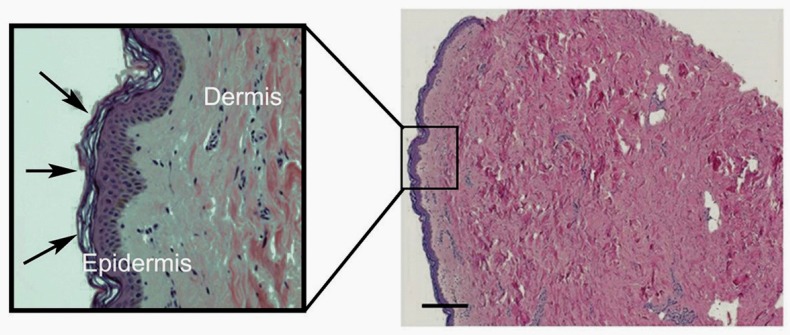
Micrograph of human breast skin sample, showing the full depth of the dermis (pink staining) in comparison to the thin layer of epidermis (purple staining). The scale bar indicates 200 µm. A zoomed-in image is shown within the box. The stratum corneum, the outermost layer of the epidermis, is indicated by the arrows, with its characteristic basket-weave structure. The collagen bundles in the dermis are very clear, as are the scattered purple-stained fibroblasts that generate this structure.

The skin is composed of two main layers with quite different underlying structures—the outermost epidermis and the deeper dermis (Figure 1). The epidermis fulfils most of the barrier functions of the skin and is predominantly made up of cells, mostly keratinocytes [2]. The keratinocytes are arranged in layers throughout the epidermis; as these cells divide and proliferate away from the basal layer, which is closest to the dermis, they begin to differentiate. This process is called keratinization, and involves the production of specialized structural proteins, secretion of lipids, and the formation of a cellular envelope of cross-linked proteins. During differentiation, virtually all of the subcellular organelles disappear, including the nucleus [3,4]. The cytoplasm is also removed, although there is evidence that some enzymes remain [4]. Thus, the uppermost layer of the epidermis that interacts with the outside environment is composed of flattened metabolically ‘dead’ cells (the terminally differentiated keratinocytes). These cells are sealed together with lipid-rich domains, forming a water-impermeable barrier. This layer is known as the stratum corneum (Figure 1) and fulfils the primary barrier function of the epidermis, although the lower epidermal layers also contribute [5].

In contrast, the dermal skin layer provides strength and elasticity, and includes the vascular, lymphatic and neuronal systems. It is relatively acellular and is primarily made up of complex extracellular matrix proteins [6], being particularly rich in collagen fibres, which make up ~75% of the dermis dry weight (Figure 1). The major cell type present in the dermis is fibroblasts, which are heavily involved in the synthesis of many of the extracellular matrix components. Blood vessels that supply nutrients to both skin layers are also present in the dermis [1,2]. Between the two main layers is the dermal–epidermal junction, a specialised basement membrane structure that fixes the epidermis to the dermis below.

## 2. Role of Nutrition in Skin Health

It is accepted that nutritional status with respect to both macronutrients and micronutrients is important for skin health and appearance [7]. Evidence of this is provided by the many vitamin deficiency diseases that result in significant disorders of the skin [8]. Dermatological signs of B vitamin deficiency, for example, include a patchy red rash, seborrhoeic dermatitis and fungal skin and nail infections [9,10]. The vitamin C deficiency disease scurvy is characterised by skin fragility, bleeding gums and corkscrew hairs as well as impaired wound healing [11,12,13,14,15,16,17,18].

Nutritional status is vital for maintaining normal functioning of the skin during collagen synthesis and keratinocyte differentiation [7]. Additionally, many of the components of our antioxidant defences such as vitamins C and E and selenium are obtained from the diet, and these are likely to be important for protection against UV-induced damage [19,20,21,22,23].

### Nutrition Issues Specific to the Skin

**Figure 2:**
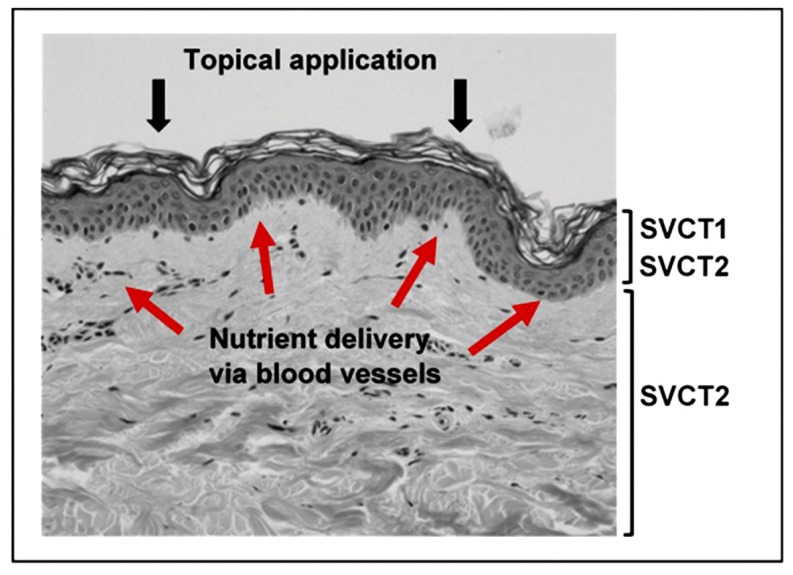
Delivery of nutrients to the skin. The location of the vitamin C transport proteins SVCT1 and SVCT2 are indicated. Red arrows depict nutrient flow from the blood vessels in the dermis to the epidermal layer. Nutrients delivered by topical application would need to penetrate the barrier formed by the stratum corneum.

The epidermis is a challenged environment for nutrient delivery, as it lacks the blood vessels that normally deliver nutrients to cells. Delivery of nutrients is dependent on diffusion from the vascularized dermis [24], and this may be particularly limited for the outermost layers of the epidermis (Figure 2). Delivery is further compounded by the chemical nature of these outer epidermal layers in which there is little movement of extracellular fluid between cells due to the complex lipid/protein crosslink structure forming the skin barrier. All of this makes it likely that dietary nutrients are not easily able to reach the cells in the outermost layers of the epidermis, and these cells receive little nutrient support.

The skin can be targeted for nutrient delivery through topical application (Figure 2). However, in this case the delivery vehicle is influential, as the stratum corneum functions as an effective aqueous barrier and prevents the passage of many substances [1]. Although some uncharged and lipid-soluble molecules can pass through the surface layer, it is unlikely that nutrients delivered via topical application would easily penetrate into the lower layers of the dermis [22]. The dermal layer functions are therefore best supported by nutrients delivered through the bloodstream.

## 3. Vitamin C Content of Skin

Normal skin contains high concentrations of vitamin C, with levels comparable to other body tissues and well above plasma concentrations, suggesting active accumulation from the circulation. Most of the vitamin C in the skin appears to be in intracellular compartments, with concentrations likely to be in the millimolar range [25,26,27]. It is transported into cells from the blood vessels present in the dermal layer. Skin vitamin C levels have not often been reported and there is considerable variation in the published levels, with a 10-fold range across a number of independent studies (Table 1). Levels are similar to that found in numerous other body organs. The variation in reported levels most likely reflects the difficulty in handling skin tissue, which is very resilient to degradation and solubilisation, but may also be due to the location of the skin sample and the age of the donor.

Several reports have indicated that vitamin C levels are lower in aged or photodamaged skin [25,26,27]. Whether this association reflects cause or effect is unknown, but it has also been reported that excessive exposure to oxidant stress via pollutants or UV irradiation is associated with depleted vitamin C levels in the epidermal layer [33,34]. Indeed, more vitamin C is found in the epidermal layer than in the dermis, with differences of 2–5-fold between the two layers being consistently reported (Table 1 and [25,26]). Levels of vitamin C in skin are similar to the levels of other water soluble antioxidants such as glutathione [25,26,27,35]. There is a suggestion that vitamin C in the stratum corneum layer of the epidermis exists in a concentration gradient [36]. The lowest vitamin C concentration was present at the outer surface of the epidermis of the SKH-1 hairless mouse, a model of human skin, with a sharp increase in concentration in the deeper layers of the stratum corneum, possibly reflecting depletion in the outer cells due to chronic exposure to the environment [36].

### 3.1. The Bioavailability and Uptake of Vitamin C into the Skin

#### 3.1.1. The Sodium-Dependent Vitamin C Transporters

Vitamin C uptake from the plasma and transport across the skin layers is mediated by specific sodium-dependent vitamin C transporters (SVCTs) that are present throughout the body and are also responsible for transport into other tissues. Interestingly, cells in the epidermis express both types of vitamin C transporter, SVCT1 and SVCT2 (Figure 2) [37]. This contrasts with most other tissues, which express SVCT2 only [37,38,39]. SVCT1 expression in the body is largely confined to the epithelial cells in the small intestine and the kidney and is associated with active inter-cellular transport of the vitamin [40,41]. The specific localisation of SVCT1 in the epidermis is of interest due to the lack of vasculature in this tissue, and suggests that the combined expression of both transporters 1 and 2 ensures effective uptake and intracellular accumulation of the vitamin. Together with the high levels of vitamin C measured in the epidermal layer, the dual expression of the SVCTs suggests a high dependency on vitamin C in this tissue.

Both transporters are hydrophobic membrane proteins that co-transport sodium, driving the uptake of vitamin C into cells. Replacement of sodium with other positively charged ions completely abolishes transport [42]. SVCT1 and SVCT2 have quite different uptake kinetics reflecting their different physiological functions. SVCT1 transports vitamin C with a low affinity but with a high capacity (K_m_ of 65–237 µmol/L) mediating uptake of vitamin C from the diet and re-uptake in the tubule cells in the kidney [41]. SVCT2, which is present in almost every cell in the body, is thought to be a high-affinity, low capacity transporter, with a K_m_ of ~20 µM meaning it can function at low concentrations of vitamin C [41]. As well as transporter affinity, vitamin C transport is regulated by the availability of the SVCT proteins on the plasma membrane.

#### 3.1.2. Bioavailability and Uptake

Most tissues of the body respond to plasma availability of vitamin C and concentrations vary accordingly, with lower tissue levels being reported when plasma levels are below saturation [43,44,45,46,47]. The kinetics of uptake varies between tissues, with vitamin C levels in some organs (e.g., the brain) reaching a plateau at lower plasma vitamin C status, whereas other tissue levels (e.g., skeletal muscle) continue to increase in close association with increasing plasma supply [32,44,45,48].

Very little is known about vitamin C accumulation in the skin and there are no studies that have investigated the relationship between skin vitamin C content and nutrient intake or plasma supply. Two human studies have shown an increase in skin vitamin C content following supplementation with vitamin C, but neither contained adequate measures of plasma vitamin C levels in the participants before or after supplementation [27,49]. In one other study, vitamin C content was measured in buccal keratinocytes, as these cells are proposed to be a good model for skin keratinocytes [50]. The keratinocyte vitamin C concentration doubled upon supplementation of the participants with 3 g/day vitamin C for six weeks, a dosage that is significantly higher than the recommended daily intake and would achieve plasma saturation and likely also tissue saturation [44].

Thus it appears likely that, as with many other tissues, skin vitamin C levels respond to increases in plasma supply [27,50]. A paper by Nusgens and co-workers suggests that skin levels do not increase further once plasma saturation is reached [51]. Dietary supplementation is therefore only expected to be effective in elevating skin vitamin C in individuals who have below-saturation plasma levels prior to intervention.

#### 3.1.3. Topical Application of Vitamin C

When plasma levels are low, some vitamin C can be delivered to the epidermal layer by topical application, although the efficacy of this is dependent on the formulation of the cream or serum used on the skin [51,52,53,54,55]. Vitamin C, as a water-soluble and charged molecule, is repelled by the physical barrier of the terminally differentiated epidermal cells. It is only when pH levels are below 4 and vitamin C is present as ascorbic acid that some penetration occurs [56], but whether this results in increased levels in the metabolically compromised stratum corneum is unknown. A great deal of effort has been put into the development of ascorbic acid derivatives for the purpose of topical application. Such derivatives need to ensure stabilization of the molecule from oxidation and also overcome the significant challenge of skin penetration. In addition, they must be converted to ascorbic acid in vivo in order to be effective. Whether there is a single solution to all these challenges is unclear [57]. The addition of a phosphate group confers greater stability and these derivatives may be converted to ascorbic acid in vivo, albeit at a slow rate [58], but they are poorly absorbed through the skin [56,59,60]. Ascorbyl glucoside also exhibits superior stability and can penetrate, but the rate of its in vivo conversion is not known [57,61,62,63]. Derivatives containing lipid-soluble moieties such as palmitate are designed to assist with delivery, and although increased uptake has been demonstrated in animals [64], they do not necessarily show improved stability and there is some doubt as to whether these derivatives are efficiently converted in vivo [57]. Recent studies suggest that encapsulation into a lipospheric form may assist with transport into the lower layers of the epidermis and could result in increased uptake [65,66,67]. However, the most pertinent issue for the efficacy of topical application is likely to be the plasma status of the individual: if plasma levels are saturated, then it appears that topical application does not increase skin vitamin C content [51].

#### 3.1.4. Vitamin C Deficiency

One of the most compelling arguments for a vital role for vitamin C in skin health is the association between vitamin C deficiency and the loss of a number of important skin functions. In particular, poor wound healing (associated with collagen formation), thickening of the stratum corneum and subcutaneous bleeding (due to fragility and loss of connective tissue morphology) are extreme and rapid in onset in vitamin-C-deficient individuals [11,15,16,17,18]. It is thought that similar processes occur when body stores are below optimal, although to a lesser extent [46,68].

## 4. Potential Functions of Vitamin C in the Skin

The high concentration of vitamin C in the skin indicates that it has a number of important biological functions that are relevant to skin health. Based on what we know about vitamin C function, attention has been focused on collagen formation and antioxidant protection; however, evidence is emerging for other activities.

### 4.1. The Promotion of Collagen Formation

**Figure 3:**
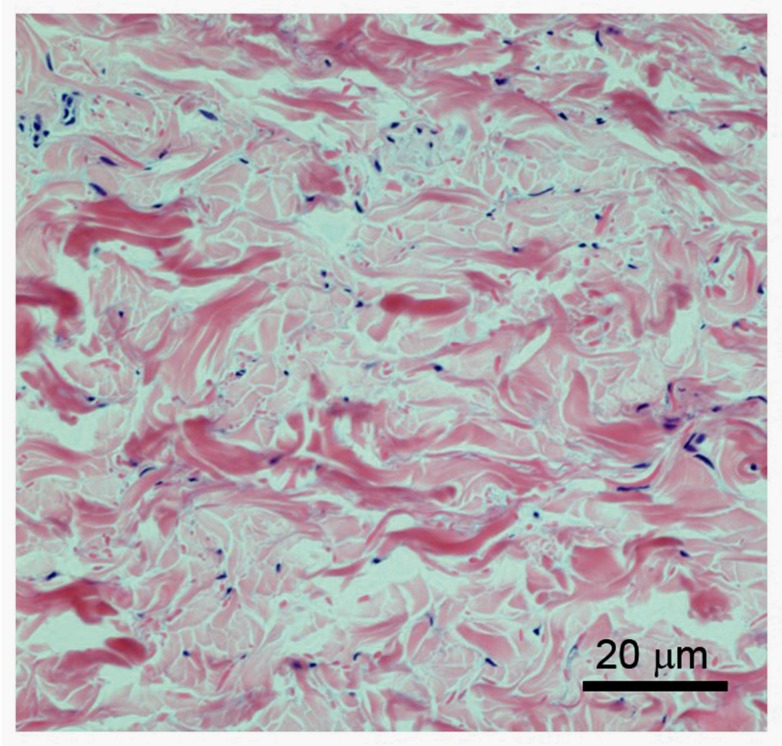
Structure of the dermis. Higher magnification of H&E-stained dermis, showing the irregular nature of the bundled collagen fibres (pink stained) and sparse presence of the fibroblasts (blue nuclear staining). Vitamin C present in the fibroblasts supports the synthesis of the collagen fibres.

Vitamin C acts as a co-factor for the proline and lysine hydroxylases that stabilise the collagen molecule tertiary structure, and it also promotes collagen gene expression [69,70,71,72,73,74,75,76,77]. In the skin, collagen formation is carried out mostly by the fibroblasts in the dermis, resulting in the generation of the basement membrane and dermal collagen matrix (Figure 3) [75,78]. The dependence of the collagen hydroxylase enzymes on vitamin C has been demonstrated in a number of studies with fibroblast cells in vitro [69,73,79], with both decreased total synthesis and decreased crosslinking when vitamin C is absent [80,81,82]. The activity of the hydroxylases is much more difficult to measure in vivo, as the amount of collagen synthesised may vary only a little [51,52]. Rather, animal studies with the vitamin-C-deficient GULO mouse indicate that the stability of the synthesised collagen varies with vitamin C availability, reflecting the stabilising function of the collagen crosslinks formed by the hydroxylases [76]. In addition to stabilising the collagen molecule by hydroxylation, vitamin C also stimulates collagen mRNA production by fibroblasts [78,83].

### 4.2. The Ability to Scavenge Free Radicals and Dispose of Toxic Oxidants

**Figure 4:**
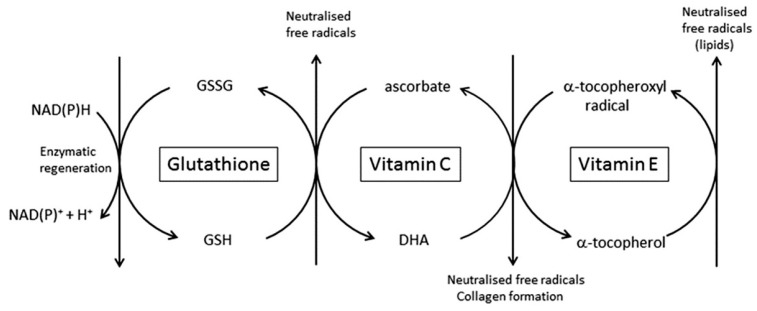
The central role for vitamin C and other antioxidants pertinent to the skin. The interdependence of vitamins E and C, and glutathione, in the scavenging of free radicals and regeneration of the reduced antioxidants, is shown. Vitamin E is in the lipid fraction of the cell, whereas vitamin C and glutathione are water-soluble and present in the cytosol.

Vitamin C is a potent antioxidant that can neutralise and remove oxidants, such as those found in environmental pollutants and after exposure to ultraviolet radiation. This activity appears to be of particular importance in the epidermis, where vitamin C is concentrated in the skin. However, vitamin C is only one player in the antioxidant arsenal that includes enzymatic defences (catalase, glutathione peroxidase and superoxide dismutase) as well as other non-enzymatic defences (vitamin E, glutathione, uric acid and other putative antioxidants such as carotenoids) [19,21,33,34,84,85,86,87,88]. Most intervention studies carried out to determine the capacity of antioxidants to prevent oxidative damage to skin have used a cocktail of these compounds [21,88,89,90]. Vitamin C is particularly effective at reducing oxidative damage to the skin when it is used in conjunction with vitamin E [21,54,89,91,92]. This is in accord with its known function as a regenerator of oxidised vitamin E, thereby effectively recycling this important lipid-soluble radical scavenger and limiting oxidative damage to cell membrane structures [92,93] (Figure 4).

### 4.3. Inhibition of Melanogenesis

Vitamin C derivatives, including the magnesium phosophate ascorbyl derivative, have been shown to decrease melanin synthesis both in cultured melanocytes and in vivo [94,95]. This activity has been proposed to be due to its ability to interfere with the action of tyrosinase, the rate-limiting enzyme in melanogenesis. Tyrosinase catalyses the hydroxylation of tyrosine to dihydroxyphenylalanine (DOPA), and the oxidation of DOPA to its corresponding ortho-quinone. The inhibition in melanin production by vitamin C is thought to be due to the vitamin’s ability to reduce the ortho-quinones generated by tyrosinase [94], although other mechanisms are also possible [96]. Agents that decrease melanogenesis are used to treat skin hyperpigmentation in conditions such as melisma or age spots.

### 4.4. Interaction with Cell Signalling Pathways

In vitro studies clearly show that vitamin C can play a role in the differentiation of keratinocytes (Table 2). For example, vitamin C enhanced the differentiation of rat epidermal keratinocytes cells in an organotypic culture model [97], with markedly improved ultrastructural organisation of the stratum corneum, accompanied by enhanced barrier function. Vitamin C also increased numbers of keratohyalin granules and levels of the late differentiation marker filaggrin, which appeared to be due to altered gene expression [97]. Others have also shown that vitamin C promotes synthesis and organization of barrier lipids and increased cornified envelope formation during differentiation [98,99,100,101,102]. The mechanism(s) by which vitamin C modulates keratinocyte differentiation is not yet elucidated; however, it has been hypothesized to be under the control of protein kinase C and AP-1 [99].

In addition to vitamin C’s ability to promote collagen synthesis [73,79], there is evidence to suggest that vitamin C increases proliferation and migration of dermal fibroblasts [78,82,102], functions vital for effective wound healing, although the underlying mechanisms driving this activity are not yet known [78]. Through the stimulation of regulatory hydroxylases, vitamin C also regulates the stabilization and activation of the hypoxia-inducible factor (HIF)-1, a metabolic sensor that controls the expression of hundreds of genes involved with cell survival and tissue remodelling, including collagenases [103,104,105]. Vitamin C has been shown to both stimulate [69] and inhibit elastin synthesis in cultured fibroblasts [81]. Glycosaminoglycan synthesis as part of extracellular matrix formation is also increased by vitamin C treatment [106], and it may also influence gene expression of antioxidant enzymes, including those involved in DNA repair [78]. As such, vitamin C has been shown to increase the repair of oxidatively damaged bases. [78]. The modulation of gene expression may be important for its ability to protect during UV exposure via its inhibition of pro-inflammatory cytokine secretion and apoptosis [107,108,109].

### 4.5. Modulation of Epigenetic Pathways

In addition to the gene regulatory activities listed above, vitamin C has a role in epigenetic regulation of gene expression by functioning as a co-factor for the ten-eleven translocation (TET) family of enzymes, which catalyse the removal of methylated cytosine through its hydroxylation to 5-hydroxymethylcytosine (5 hmC) [110,111,112]. As well as being a DNA demethylation intermediate, it appears that 5 hmC is an epigenetic mark in its own right, with transcriptional regulatory activity [113]. Aberrant epigenetic alterations are thought to have a role in cancer progression, and there is data to suggest that a loss of 5 hmC occurs during the early development and progression of melanoma [114]. Interestingly, vitamin C treatment has been shown to increase 5 hmC content in melanoma cell lines, also causing a consequent alteration in the transcriptome and a decrease in malignant phenotype [115]. Because TETs have a specific requirement for vitamin C to maintain enzyme activity [116], this provides a further mechanism by which the vitamin may affect gene expression and cell function. For example, Lin and co-workers showed that vitamin C protected against UV-induced apoptosis of an epidermal cell line via a TET-dependent mechanism, which involved increases in p21 and p16 gene expression [117].

## 5. Challenges to the Maintenance of Skin Health and Potential Protection by Vitamin C

During the course of a normal lifetime, the skin is exposed to a number of challenges that can affect structure, function and appearance, including:

- Deterioration due to normal aging, contributing to loss of elasticity and wrinkle formation.
- Exposure to the elements, leading to discolouration, dryness and accelerated wrinkling.
- Chemical insults including exposure to oxidising beauty and cleansing products (hair dyes, soaps, detergents, bleaches).
- Direct injury, as in wounding and burning.

Vitamin C may provide significant protection against these changes and regeneration of healthy skin following insult and injury is a goal for most of us. The following sections, and the summary in Table 3 and Table 4, review the available evidence of a role for vitamin C in the maintenance of healthy skin and the prevention of damage.

### 5.1. Skin Aging

Like the rest of the human body, the skin is subject to changes caused by the process of natural aging. All skin layers show age-related changes in structure and functional capacity [6,120] and, as occurs in other body systems, this may result in increased susceptibility to a variety of disorders and diseases, such as the development of dermatoses and skin cancer [6,22,121,122]. As well as this, changes in the appearance of skin are often the first visible signs of aging and this can have implications for our emotional and mental wellbeing.

Aging of skin can be thought of as two distinct processes—natural or ‘intrinsic’ aging, caused simply by the passage of time, and environmental aging [121,123,124]. Lifestyle factors such as smoking and exposure to environmental pollutants increase the rate of environmental aging, and can have a marked impact on the function and appearance of skin [22,121,122,123,124]. Exposure to chronic ultraviolet radiation from sunlight is also a major environmental factor that prematurely damages our skin (effects are detailed in the photoaging section below) [125]. The changes due to environmental aging are usually superimposed on those that occur naturally, often making it difficult to distinguish between the two [22].

Intrinsic aging is a slow process and, in the absence of environmental aging, changes are not usually apparent until advanced age, when smooth skin with fine wrinkles, pale skin tone, reduced elasticity, and occasional exaggerated expression lines are evident [6,22,24]. There is a reduction in the thickness of the dermal layer [22], along with fewer fibroblasts and mast cells, less collagen production and reduced vascularisation [24]. Specifically, during intrinsic aging there is gradual degradation of the extracellular matrix components, particularly elastin and collagen [124,126]. The loss of elastin results in the reduction in elasticity and capacity for recoil that is observed in aging skin.

Dry skin is very common in older adults [127], largely due to a loss of glycosaminoglycans and accompanied reduction in the ability to maintain moisture levels [126,128]. The dermal-epidermal junction may also become flattened, losing surface area and leading to increased skin fragility [22], and potentially causing reduced nutrient transfer between the two layers. In general, the dermis suffers from greater age-related changes than the epidermis [1]. However, the aged epidermis shows a reduced barrier function and also reduced repair following insult [6]. Antioxidant capacity, immune function and melanin production may also be impaired in aged skin [22].

Intrinsic aging is largely unavoidable and may be largely dependent on our genetic background and other factors [129,130]. Some mitigation of these effects may be achieved by:

- Limiting exposure to environmental risk factors such as smoking, poor nutrition and chronic exposure to sunlight, which cause premature skin aging.
- Using treatments to potentially reverse skin damage, including topical or systemic treatments that help regenerate the elastic fibre system and collagen [126].

#### 5.1.1. The Role of Vitamin C in the Prevention of Skin Aging

The ability of vitamin C to limit natural aging is difficult to distinguish from its ability to prevent the additional insults due to excessive sun exposure, smoking or environmental stress and there is very limited information available concerning a relationship between vitamin C levels and general skin deterioration. The most compelling argument for a role of vitamin C in protecting skin function comes from observations that deficiency causes obvious skin problems—early signs of scurvy, for example, include skin fragility, corkscrew hairs and poor wound healing [11,12,13,14,15,16,17].

Because vitamin C deficiency results in impaired function, it is assumed that increasing intake will be beneficial. However, there are no studies that have measured vitamin C levels or intake and associated aging changes [130]. Vitamin C is almost never measured in the skin and this information is needed before we can improve our understanding of what level of intake might be beneficial for skin health and protection against aging-related changes.

#### 5.1.2. Nutritional Studies Linking Vitamin C with Skin Health

Although there is no information specific to vitamin C and aging in the skin, many studies have attempted to determine the role of nutrition more generally [85,131,132,133]. A recent systematic review of studies involving nutrition and appearance identified 27 studies that were either dietary intervention studies or reported dietary intakes [134]. The analysis indicated that, in the most reliable studies, intervention with a nutrient supplement (15 studies) or general foods (one study) was associated with improved measures of skin elasticity, facial wrinkling, roughness and colour [134]. Many of the nutrient interventions that showed a benefit included a high intake of fruit and vegetables, which contribute significant levels of vitamin C to the diet.

A double-blind nutrition intervention study has evaluated the effects of dietary supplementation with a fermented papaya extract, thought to have antioxidant activity [135], and an antioxidant cocktail containing 10 mg trans resveratrol, 60 μg selenium, 10 mg vitamin E and 50 mg vitamin C in a population of healthy individuals aged between 40 and 65, all with visible signs of skin aging. Following a 90-day supplementation period, skin surface, brown spots, evenness, moisture, elasticity (face), lipid peroxidation markers, superoxide dismutase activity, nitric oxide (NO) concentration, and the expression levels of key genes were measured. Notably, the intervention resulted in a measureable improvement in skin physical parameters, with a generally enhanced response from the fermented papaya extract compared with the antioxidant cocktail. Gene expression, measured by RNA extraction and RT-PCR, indicated that the papaya extract increased expression of aquaporin-3, and decreased expression of cyclophilin A and CD147. Aquaporin 3 regulates water transport across the lipid bilayer in keratinocytes and fibroblasts and therefore improves skin health [136]; cyclophilin A and the transmembrane glycoprotein CD147 negatively impact on skin DNA repair mechanisms and affect the inflammatory response, therefore negatively impacting skin health. This is an interesting study and suggests that antioxidant supplementation, including vitamin C, could benefit skin health generally. The antioxidant cocktail did not affect gene expression, and this may reflect the low concentrations of each component in the supplement, which is unlikely to influence levels in a healthy population. Although there were no direct measures to determine whether antioxidant status was actually improved in the participants, antioxidant activity was improved in the skin following intake of the papaya extract, as evidenced by decreased markers of lipid peroxidation and increased superoxide dismutase activity.

### 5.2. UV Radiation and Photoaging

There is mounting evidence to suggest that the most significant environmental challenge to the skin is chronic exposure to ultraviolet radiation from the sun or from tanning beds [22,90,123,137]. UV radiation damages skin through the production of reactive oxygen species, which can damage the extracellular matrix components and affect both the structure and function of cells. While the skin contains endogenous antioxidant defences, vitamins E and C and antioxidant enzymes to quench these oxidants and repair the resultant damage, these antioxidants will be consumed by repeated exposure and the skin’s defences can thereby be overwhelmed [25,138,139,140,141].

Acute exposure of skin to UV radiation can cause sunburn, resulting in a large inflammatory response causing characteristic redness, swelling and heat. In addition, altered pigmentation, immune suppression and damage to the dermal extracellular matrix can occur [24,25,56,142,143]. By comparison, chronic long-term exposure to UV radiation causes premature aging of the skin, with dramatic and significant disruption to skin structure, and leads to the development of skin cancer [6,24]. Termed photoaging, the most obvious features are wrinkles, hyperpigmentation and marked changes in skin elasticity that cause skin sagging, with the skin also becoming sallow and rougher with age [123,144]. Photoaged skin is most likely to be found on the face, chest and upper surface of the arms.

Both the epidermal and dermal layers of skin are susceptible to chronic UV exposure; however, the most profound changes occur in the extracellular matrix of the dermis [24]. Changes include a significant loss of collagen fibrils within the dermis, but also specific loss of collagen anchoring fibrils at the dermal–epidermal junction [126]. Dermal glycosaminoglycan content is increased in what appear to be disorganised aggregates [126]. Elastic fibres throughout the dermis are also susceptible to UV radiation, with accumulation of disorganised elastic fibre proteins evident in severely photoaged skin. Indeed, this accumulation, termed ‘solar elastosis’, is considered to be a defining characteristic of severely photoaged skin [6,22,24,126]. There is also evidence of epidermal atrophy or ‘wasting away’ during photoaging, and of a reduction in the barrier function [6]. In addition, the epidermis can become hyperpigmented from chronic UV exposure; these lesions are known as age spots or liver spots.

Preventing exposure to UV radiation is the best means of protecting the skin from the detrimental effects of photoaging. However, avoidance is not always possible, so sunscreen is commonly used to block or reduce the amount of UV reaching the skin. However, sunscreens expose the skin to chemicals that may cause other problems such as disruption of the skin barrier function or induction of inflammation [56].

#### Vitamin-C-Mediated Protection against Photoaging and UV Damage

Changes to the skin due to UV exposure have much in common with the rather slower process of ‘natural’ aging, with one major difference being a more acute onset. It is established that vitamin C limits the damage induced by UV exposure [27,54,89,145,146]. This type of injury is directly mediated by a radical-generating process, and protection is primarily related to its antioxidant activity. This has been demonstrated with cells in vitro and in vivo, using both topical and dietary intake of vitamin C [54,139,147,148]. It appears that UV light depletes vitamin C content in the epidermis, which also indicates that it is targeted by the oxidants induced by such exposure [138,149]. Vitamin C prevents lipid peroxidation in cultured keratinocytes following UV exposure and also protects the keratinocyte from apoptosis and increases cell survival [21,99,107].

Sunburn is measured as the minimal erythemal dose (MED) in response to acute UV exposure. A number of studies have shown that supplementation with vitamin C increases the resistance of the skin to UV exposure. However, vitamin C in isolation is only minimally effective, and most studies showing a benefit use a multi-component intervention [21,50,86,90,107,150,151,152]. In particular, a synergy exists between vitamin C and vitamin E, with the combination being particularly effective [50]. These results indicate the need for complete oxidant scavenging and recycling as indicated in Figure 4, in order to provide effective protection from UV irradiation. This combination also decreases the inflammation induced by excessive UV exposure.

Topical application of vitamin C, in combination with vitamin E and other compounds, has also been shown to reduce injury due to UV irradiation [50,54,65,89,150,152,153]. However, the efficacy of topical vitamin C and other nutrients may depend on the pre-existing status of the skin. One study suggests that when health status is already optimal there is no absorption of vitamin C following topical application. Hence, “beauty from the inside”, via nutrition, may be more effective than topical application [132].

Vitamin-C-mediated prevention of radiation injury from acute UV exposure is relatively easily demonstrated, and these studies are highlighted above. However, reversal of photoaging due to prior, chronic sun damage is much more problematic. Although there are a number of studies that claim a significant benefit from an antioxidant supplement or topical cream, interpretation of the data is confounded by the complex formulation of the interventions, with most studies using a cocktail of compounds and with the formulation of topical creams providing a moisturising effect in itself [23,61,88,154].

### 5.3. Dry Skin

Dry skin is a common condition typically experienced by most people at some stage in their lives. It can occur in response to a particular skin care regime, illness, medications, or due to environmental changes in temperature, air flow and humidity. The prevalence of dry skin also increases with age [127]; this was originally believed to be due to decreased water content or sebum production in the skin as we get older. However, it is now considered likely to be due to alterations in the keratinisation process and lipid content of the stratum corneum [127].

The pathogenesis of dry skin is becoming clearer and three contributing deficiencies have been identified.

- A deficiency in the skin barrier lipids, the ceramides, has been identified. These lipids are the main intercellular lipids in the stratum corneum, accounting for 40 to 50 percent of total lipids [155].
- A reduction in substances known as the natural moisturising factor (NMF) [156,157] is also thought to be involved in dry skin. These substances are found in the stratum corneum within the corneocytes, where they bind water, allowing the corneocyte to remain hydrated despite the drying effects of the environment.
- More recently, a deficiency of the skin’s own moisture network in the epidermis, mediated by the newly discovered aquaporin water channels, has been suggested to play a role [131].

Treatment of dry skin involves maintenance of the lipid barrier and the natural moisturising factor components of the stratum corneum, generally through topical application (91), although nutritional support of the dermis may also be useful [135,156].

#### Potential for Vitamin C to Prevent Dry Skin Conditions

Cell culture studies have shown that the addition of vitamin C enhances the production of barrier lipids and induces differentiation of keratinocytes, and from these observations it has been proposed that vitamin C may be instrumental in the formation of the stratum corneum and may thereby influence the ability of the skin to protect itself from water loss [99,157]. Some studies have indicated that topical application of vitamin C may result in decreased roughness, although this may depend more on the formulation of the cream than on the vitamin C content [52,55]. Because most studies in this area involve topical application, the complex and variable effects (pH and additional compounds) of topical formulations make it difficult to come to any firm conclusion as to whether vitamin C affects skin dryness.

### 5.4. Wrinkles

Wrinkles are formed during chronological aging and the process is markedly accelerated by external factors such as exposure to UV radiation or smoking. The formation of wrinkles is thought to be due to changes in the lower, dermal layer of the skin [22] but little is known about the specific molecular mechanisms responsible. It is thought that loss of collagen, deterioration of collagen and elastic fibres and changes to the dermal–epidermal junction may contribute [22,120,158,159,160]. One hypothesis is that UV light induces cytokine production, which triggers fibroblast elastase expression causing degradation of elastic fibres, loss of elasticity and consequent wrinkle formation.

#### The Effect of Vitamin C on Wrinkle Formation and Reversal

The appearance of wrinkles, or fine lines in the skin, has a major impact on appearance and is therefore often a focus of intervention studies. Most have used topical applications, generally containing a mixture of vitamin C and other antioxidants or natural compounds, with varied efficacy [51,52,161]. Generally the demonstration of wrinkle decrease in these studies is less than convincing, and the technology to measure these changes is limited. More recently, improved and impartial imaging technologies such as ultrasound have been used to determine the thickness of the various skin layers [135,149]. Once again, the efficacy of topical vitamin C creams on wrinkled skin may depend on the vitamin C status of the person involved. An indication that improved vitamin C status could protect against wrinkle formation through improved collagen synthesis comes from the measured differences in wound healing and collagen synthesis in smokers, abstinent smokers and non-smokers with associated variances in plasma vitamin C status [162]. Smokers had depleted vitamin C levels compared with non-smokers; these levels could be improved by smoking cessation, with an associated improvement in wound healing and collagen formation [162].

### 5.5. Wound Healing

Wound healing is a complex process with three main consecutive and overlapping stages; inflammation, new tissue formation and remodelling [163]. Following vasoconstriction and fibrin clot formation to stem bleeding, inflammatory cells are recruited to the wound site. The first of these cells is the neutrophil, which clears the wound of any damaged tissue and infectious material and signals the recruitment of tissue macrophages [164]. Macrophages continue clearing damaged material and bacteria, including spent neutrophils. Crucially, they are thought to be involved in orchestrating the healing process, signalling fibroblasts to remodel tissue at the wound site and providing vital signals for re-epithelialisation and dermal repair [163,164].

Re-epithelisation restores the skin’s barrier function, and occurs by a combination of migration and proliferation of the epidermal keratinocytes that reside close to the damaged area. Epidermal stem cells may also be involved in re-epithelisation [163]. In addition to the epidermal layer, the underlying dermis must also be restored. Fibroblasts from a number of sources also proliferate and move into the wound area [165], where they synthesise extracellular matrix components. These cells remove the fibrin clot from the wound area, replacing it with a more stable collagen matrix. They are also involved in wound contraction, and the reordering of collagen fibres. Proliferation of blood vessels is initiated by growth factor production by macrophages, keratinocytes and fibroblasts.

Typically, the final result of the healing process is the formation of a scar. This is an area of fibrous tissue generally made up of collagen arranged in unidirectional layers, rather than the normal basket-weave pattern. As such, the strength of skin at the repair site is never as great as the uninjured skin [163]. Variations in scar formation can occur, resulting in keloids—raised and fibrous scars—or weak thin scar tissue. At this stage no intervention has been able to prevent the formation of scar tissue although the extent of scarring may be ameliorated [166]. It is thought that nutritional support for regeneration of the skin layers is important for development of strong healthy skin [167].

#### Vitamin C and the Benefits for Wound Healing

Of all effects of vitamin C on skin health, its beneficial effect on wound healing is the most dramatic and reproducible. This is directly related to its co-factor activity for the synthesis of collagen, with impaired wound healing an early indicator of hypovitaminosis C [68,168]. Vitamin C turnover at wound sites, due to both local inflammation and the demands of increased collagen production, means that supplementation is useful, and both topical application and increased nutrient intake have been shown to be beneficial [166,169,170]. Supplementation with both vitamin C and vitamin E improved the rate of wound healing in children with extensive burns [171], and plasma vitamin C levels in smokers, abstaining smokers and non-smokers were positively associated with the rate of wound healing [162]. However, it would appear that the extent of the benefits of supplemented vitamin C intake is, once again, dependent upon the status of the individual at baseline, with any benefit being less apparent if nutritional intake is already adequate [167,168]. However, the complexity and poor selection of study population has often made it difficult to come to firm conclusions about the efficacy of nutritional interventions, as summarised in a meta-analysis of the effects of varied treatments on ulcer healing [172]. In a recent study, topical application of vitamin C in a silicone gel resulted in a significant reduction in permanent scar formation in an Asian population [166].

### 5.6. Skin Inflammatory Conditions

Inflammation in the skin underlies a number of debilitating conditions such as atopic dermatitis, psoriasis and acne, with symptoms including pain, dryness and itching. The pathology underlying these conditions is complex and involves activation of auto-immune or allergic inflammation with associated generation of cytokines and cellular dysfunction, and consequent breakdown of the skin epidermal lipid barrier [173,174]. Treatments are therefore targeted at both the underlying inflammation and the repair and maintenance of the epidermal structures. Nutrition plays an integral part in both these aspects and numerous studies have investigated the impact of dietary manipulation for alleviation of acute and chronic skin pathologies, although firm conclusions as to efficacy remain elusive [175,176,177]. Treatments involving supplementation with essential omega-fatty acids, lipid-soluble vitamins E and A are often employed in an attempt to assist the generation of the lipid barriers and to retain moisture in the skin [177]. Vitamin C is often used in anti-inflammatory formulations or as a component of nutrition studies but its individual efficacy has not been investigated [175,176,177].

#### Vitamin C and Skin Inflammation

Vitamin C status has been reported to be compromised in individuals with skin inflammation, with lower levels measured compared with unaffected individuals [178,179]. This may reflect increased turnover of the redox-labile vitamin C, as is seen in many inflammatory conditions [180,181,182], and decreased vitamin C status could be expected to impact on the numerous essential functions for which it is essential as detailed in the sections above. Recent studies have begun to provide more detailed information as to specific functional implications for suboptimal vitamin C status in inflamed skin lesions. One notable study [179] has reported significantly compromised vitamin C status in patients with atopic dermatitis, with plasma levels ranging between 6 and 31 μmol/L (optimal healthy levels > 60 µM), and an inverse relationship between plasma vitamin C and total ceramide levels in the epidermis of the affected individuals. As indicated in the sections above, ceramide is the main lipid of the stratum corneum and its synthesis involves an essential hydroxylation step catalysed by ceramide synthase, an enzyme with a co-factor requirement for vitamin C [100]. Hence the potential impact of vitamin C extends far beyond its capacity as an inflammatory antioxidant in a pathological setting.

## 6. Conclusions

The role of vitamin C in skin health has been under discussion since its discovery in the 1930s as the remedy for scurvy. The co-factor role for collagen hydroxylases was the first vitamin C function that was closely tied to the symptoms of scurvy and the realisation of the importance of this function for the maintenance of skin health throughout the human lifespan led to the hypothesised skin health benefit of vitamin C. In addition, the antioxidant activity of vitamin C made it an excellent candidate as a protective factor against UV irradiation. These two hypotheses have driven most of the research into the role of vitamin C and skin health to date.

The following information is available as a result of research into the role of vitamin C in skin health, and Table 2 and Table 4 list a sample of key studies:

- Skin fibroblasts have an absolute dependence on vitamin C for the synthesis of collagen, and for the regulation of the collagen/elastin balance in the dermis. There is ample in vitro data with cultured cells demonstrating this dependency. In addition, vitamin C supplementation of animals has shown improved collagen synthesis in vivo.
- Skin keratinocytes have the capacity to accumulate high concentrations of vitamin C, and this in association with vitamin E affords protection against UV irradiation. This information is available from in vitro studies with cultured cells, with supportive information from animal and human studies.
- Analysis of keratinocytes in culture has shown that vitamin C influences gene expression of antioxidant enzymes, the organisation and accumulation of phospholipids, and promotes the formation of the stratum corneum and the differentiation of the epithelium in general.
- Delivery of vitamin C into the skin via topical application remains challenging. Although some human studies have suggested a beneficial effect with respect to UV irradiation protection, most effective formulations contain both vitamins C and E, plus a delivery vehicle.
- Good skin health is positively associated with fruit and vegetable intake in a number of well-executed intervention studies. The active component in the fruit and vegetables responsible for the observed benefit is unidentified, and the effect is likely to be multi-factorial, although vitamin C status is closely aligned with fruit and vegetable intake.
- Signs of aging in human skin can be ameliorated through the provision of vitamin C. A number of studies support this, although measurement of skin changes is difficult. Some studies include objective measures of collagen deposition and wrinkle depth.
- The provision of vitamin C to the skin greatly assists wound healing and minimises raised scar formation. This has been demonstrated in numerous clinical studies in humans and animals.

## Figures and Tables

**Table 1:** Vitamin C content of human skin and a comparison with other tissues.

Tissue	Vitamin C Content (mg/100 g Wet Weight)	References
Adrenal glands	30–40	[28]
Pituitary glands	40–50	[29]
Liver	10–16	[28,30]
Spleen	10–15	[28,31]
Lungs	7	[28]
Kidneys	5–15	[30]
Heart muscle	5–15	[28,29,31]
Skeletal muscle	3–4	[29,32]
Brain	13–15	[28]
Skin-epidermis	6–64	[25,26,27]
Skin-dermis	3–13	[25,26,27]

**Table 2:** Summary of key in vitro studies investigating potential effects of vitamin C on the skin.

Study Description	Measured Parameters	Outcome and Comment	Reference
**Effects on collagen and elastin synthesis**			
Vit. C effects on collagen and elastin synthesis in human skin fibroblasts and vascular smooth muscle cells.	Monitored vit. C time of exposure and dose on collagen synthesis and gene expression, and elastin synthesis and gene regulation.	Vit. C exposure increased collagen, decreased elastin. Stabilization of collagen mRNA, lesser stability of elastin mRNA, and repression of elastin gene transcription.	[81]
Effect of vit. C on collagen synthesis and SVCT2 expression in human skin fibroblasts. Vit. C added to culture medium for 5 days.	Vit. C uptake measured into cells, collagen I and IV measured with RT-PCR and ELISA, and RT-PCR for SVCT2.	Vit. C increased collagen I and IV, and increased SVCT2 expression.	[73]
Effect of vit. C on elastin generation by fibroblasts from normal human skin, stretch-marked skin, keloids and dermal fat.	Immunohistochemistry and western blotting for detection of elastin and precursors.	50 and 200 µM vit. C increased elastin production, 800 µM inhibited. No measures of vit. C uptake into cells.	[69]
**Effects on morphology, differentiation and gene expression**			
Vit. C addition to cultures of rat keratinocytes (REK).	Effect on differentiation and stratum corneum formation.	Morphology showed enhanced stratum corneum structure, increased keratohyalin granules and organization of intercellular lipid lamellae in the interstices of the stratum corneum. Increased profilaggrin and filaggrin.	[97]
Effect of vit. C on human keratinocyte (HaCaT) cell line differentiation in vitro.	Measured development of cornified envelope (CE), gene expression.	CE formation and keratinocyte differentiation induced by vit. C, suggesting a role in formation of stratum corneum and barrier formation in vivo.	[99]
Effect of vit. C supplementation on gene expression in human skin fibroblasts.	Total RNA nano assay, for genetic profiling, with and without vit. C in culture medium.	Increased gene expression for DNA replication and repair and cell cycle progression. Increased mitogenic stimulation and cell motility in the context of wound healing. Faster repair of damaged DNA bases.	[78]
Effect of vit. C on dermal epidermal junction in skin model (keratinocytes and fibroblasts).	Keratinocyte organisation, fibroblast number, basement membrane protein deposition and mRNA expression.	Vit. C improved keratinocyte and basement membrane organisation. Increased fibroblast number, saw deposition of basement membrane proteins.	[102]
Effect of vit. C on cultured skin models—combined human epidermal keratinocytes and dermal fibroblasts.	Monitored morphology, lipid composition.	Vit. C, but not vit. E, improved epidermal morphology, ceramide production and phospholipid layer formation.	[98]
**Protective effects against UV irradiation**			
Effect of vit. C on UVA irradiation of primary cultures of human keratinocytes.	Vit. C added in low concentrations, monitored MDA, TBA, GSH, cell viability, IL-1, IL-6 generation.	Vit. C improved resistance to UVA, decreased MDA and TBA levels, increased GSH levels, decreased IL-1 and IL-6 levels.	[109]
Effect of vit. C uptake into human keratinocyte (HaCaT) cell line on outcome to UV irradiation.	Accumulation of vit. C in keratinocytes, antioxidant capacity by DHDCF and apoptosis induction by UV irradiation.	Keratinocytes accumulated mM levels of vit. C, increasing antioxidant status and protecting against apoptosis.	[108]
Effect of UVB on vit. C uptake into human keratinocyte cell line (HaCaT) and effects on inflammatory gene expression.	Cellular vit. C measured by HPLC, mRNA expression for chemokines, western blotting for SVCT localisation.	Vit. C uptake was increased with UVB irradiation, chemokine expression decreased with vit. C uptake.	[107]
**Protective effects against ozone exposure**			
Effect of antioxidant mixtures of vit. C, vit. E and ferulic acid on exposure of cultured normal human keratinocytes to ozone.	Cell viability, proliferation, HNE, protein carbonyls, Nrf2, NFkappaB activation, IL-8 generation.	Vit. C-containing mixtures inhibited toxicity. The presence of vit. E provided additional protection against HNE and protein carbonyls.	[118]
Protection of cultured skin cells against ozone exposure with vit. C, vit. E, and resveratrol. 3-D culture of human dermis—fibroblasts with collagen I + III.	Cell death, HNE levels, expression of transcription factors Nrf-2 and NfkappaB	Extensive protection against cell damage with mixtures containing vit. C. Increased expression of antioxidant proteins. Additional effect of vit. E + C. No effect with Vit. E alone.	[119]

**Table 3:** Skin ailments, their causes and evidence from in vitro and in vivo studies for association with vitamin C levels.

Type of Skin Damage	Cause	Skin Structure Affected	Evidence of Protection by Vitamin C	References
Sunburn	Acute and excessive UV exposure.	Cell death of all skin cells, with associated inflammation.	Improving skin vitamin C and vitamin E levels can improve resistance to UV exposure.	[21,50,86,90,107,150,151,152]
Photoaging, oxidant-induced damage	Chronic UV overexposure, cigarette smoking.	Damaged collagen and elastin matrix, thinning of the epidermal layer.	Decreased signs of aging with higher fruit and vegetable intake. Protection inferred from studies with acute UV exposure.	[27,54,89,139,145,146,147,148]
Hyperpigmentation	Chronic UV exposure and environmental stresses.	Excessive pigment formation and propagation of melanocytes in the epidermis.	Nutrition studies showing improved skin colour with higher fruit and vegetable intake.	Reviewed in [134,135]
Wrinkle formation	Natural aging, oxidative stress, UV exposure, smoking, medical treatments.	Dermal layer changes, deterioration of collagen and elastic fibres.	Lessening of wrinkle depth following vitamin C supplementation. Increased collagen formation by fibroblasts in cell culture.	[69,73,79,80,81,82,135,149]
Skin sagging	Natural aging, oxidative stress damage, extreme weight loss.	Loss of elastin and collagen fibres, thinning of skin layers, loss of muscle tone.	Improved skin tightness in individuals with higher fruit and vegetable intake.	Reviewed in [134,135]
Loss of colour	Natural aging, UV exposure, illness.	Thinning of skin layers, loss of melanocytes or decreased melanin formation, loss of vasculature in dermis.	Improved skin tone with high fruit and vegetable intake.	Reviewed in [94,95,134,135]
Surface roughness	Chemical and UV exposure, physical abrasion, allergy and inflammation.	Stratum corneum, loss of skin moisture barrier function.	Vitamin C enhances production of barrier lipids in cell culture.	[98,99,100,101,102,157]
Dry skin	Medications, illness, extreme temperature, low humidity and wind exposure.	Stratum corneum, loss of skin barrier lipids and natural moisturising factor.	Vitamin C enhances production of barrier lipids in cell culture.	[98,99,100,101,102,157]
Excessive scar formation, generation of keloids	Ineffective wound healing.	Fibroblast function, collagen and elastin formation.	Supplementation improves wound healing, prevents keloid formation in vivo, enhances collagen formation by fibroblasts in vitro.	[73,79,80,81,82,166,167]
Poor wound healing, thickening rough skin	Vitamin C deficiency.	All skin cell functions, collagen formation.	Direct association Vitamin C deficiency prevents wound healing.	[162,166,169]
Inflammatory skin lesions	Allergic and auto-inflammation.	Skin barrier integrity, underlying inflammation and swelling.	Nutrition support, decreased levels associated with loss of barrier lipid ceramide.	[179]

**Table 4:** Summary of key and recent in vivo studies providing evidence of vitamin C effects in the skin.

Study Description	Measured Parameters	Outcome and Comment	References
**Animal Studies**			
**Oral Supplementation**			
Dietary supplementation of pregnant female rats. Addition of 1.25 mg/mL vitamin C to drinking water for duration of gestation.	Monitored collagen and elastin content of uterosacral ligaments by histology staining and subjective assessment.	Increased collagen production in vit.- C-supplemented rats, decreased elastin loss. Implied prevention of pelvic organ prolapse and stress urinary incontinence.	[183]
Wound healing in guinea pigs following supplementation with moderate and high-dose vit. C.	Dorsal wound healing rate and strength of repair monitored.	Increased vit. C associated with faster wound recovery and strength of skin integrity. Small sample size limited stats.	[184]
**Topical application**			
Topical application of vit. C and vit. E-containing cream to nude mice, followed by UV irradiation.	Measured melanocyte differentiation post-irradiation. Change of skin colour—tanning, inflammation.	UVR-induced proliferation and melanogenesis of melanocytes were reduced by vit. C and E. Melanocyte population and confluence reduced when vit. C present.	[185]
Cultured skin—human keratinocytes and fibroblasts attached to collagen-glycosamino-glycan substrates, incubated for five weeks ± 0.1 mM vit. C, and then grafted to athymic mice.	Collagen IV, collagen VII and laminin 5 synthesis, epidermal barrier formation and skin graft take in athymic nude mice.	Increased cell viability and basement membrane development in vitro, better graft ability in vivo.	[157]
**Human Studies**			
**Oral supplementation**			
90-day oral supplementation with a fermented papaya preparation or an antioxidant cocktail (10 mg trans-resveratrol, 60 μg selenium, 10 mg vitamin E, 50 mg vitamin C) in 60 healthy non-smoker males and females aged 40–65 years, all with clinical signs of skin aging.	Skin surface, brown spots, skin evenness, skin moisture, elasticity (face), lipid peroxidation, superoxide dismutase levels, nitric oxide (NO) generation, and the expression levels of key genes (outer forearm sample).	Improved skin elasticity, moisture and antioxidant capacity with both fermented papaya and antioxidant cocktail. Increased effect of papaya extract and on gene expression. No baseline measures in study population. Antioxidant components of the fermented papaya unknown and direct link with vit. C not available.	[135]
Intervention with 47 men aged 30–45 given oral supplement of 54 mg or 22 mg of vit. C, 28 mg tomato extract, 27 mg grape seed extract, 210 mg of marine complex, 4 mg zinc gluconate for 180 days.	Subjective assessment of appearance and objective measures of collagen and elastin (histology and measurement in biopsy material).	Improvement in erythema, hydration, radiance, and overall appearance. Decreased intensity of general skin spots, UV spots, and brown spots, improved skin texture and appearance of pores. Increased collagen (43%–57%) and elastin (20%–31%).	[49]
Supplementation of 33 healthy men and women (aged 22–50), with placebo, 100 mg vit. C or 180 mg vit. C daily for four weeks.	EPR measurement of TEMPO scavenging in skin on arm. Raman resonance spectroscopy for skin carotenoids.	Improved oxygen radical scavenging with vit. C supplementation, dose dependency indicated and rapid response (obvious within two weeks).	[38]
Three month supplementation of 12 males and six females (21–77 y) with 2 g vit. C and 1000 IU D-alpha-tocopherol.	Measured blood vitamin levels before and after, skin resilience to UVB, detection of DNA crosslinks in skin biopsy.	Serum vit. C and vit. E doubled during intervention (implies sub-saturation at baseline). Minimal erythema dose increased with supplementation, DNA damage halved.	[20]
Investigation of antioxidant capacity in human skin before and after UV irradiation; effect of supplementation with 500 mg vit. C per day.	Measurement of erythema and antioxidant levels following UVB irradiation.	Vit. C and E levels increased, but levels not realistic (plasma vit. C 21 µM before and 26 µM after 500 mg daily). Skin MDA and glutathione content lowered, no effect on MED.	[27]
**Topical application**			
Topical application of vit. C cream in advance of application of hair dye product p-phenylenediamine.	Visual assessment of allergic reaction following patch application on volunteer skin (on back).	Decreased or ablation of dermatitis and allergic response due to local antioxidant action of vit. C in cream.	[170]
Clinical study applying vit. C in liposomes to human skin (abdomen), then exposure to UV irradiation.	Measured penetration through skin layers, delivery of vit. C, loss of Trolox, TNFalpha and Il-1beta.	Increased vit. C levels in epidermis and dermis with liposomes. Protection against UV increased over liposomes alone.	[67]
Microneedle skin patches to deliver vit. C into the skin assessed on areas of slight wrinkle formation (around eyes).	Global Photodamage Score by visual inspection. Skin replica analysis and skin assessment by visiometer.	Slightly improved photodamage score and lessening of wrinkles after 12 weeks of treatment with vit. C-loaded patches.	[186]
Vit. C-based solution containing Rosa moschata oil rich in vitamins A, C, E, essential fatty acids /placebo moisturizer cream applied to facial skin of 60 healthy female subjects for 40–60 days.	Ultrasound monitoring thickness of the epidermis and dermis, and low (LEP), medium (MEP), high echogenic pixels (HEP), reflecting hydration, inflammatory processes, elastin and collagen degeneration (LEP), and structure of collagen, elastin and microfibrils (MEP and LEP).	Data suggest epidermis but not the dermis increased in thickness. Increase in MEP and HEP (collagen and elastin synthesis) and decreased LEP (inflammation and collagen degeneration). No vit. C status measurements in skin of individuals.	[149]
In vivo study with 30 healthy adults. Protective effect of SPF30 sunscreen with and without anti-oxidants (vit. E, grape seed extract, ubiquinone and vit. C) against Infra-Red A irradiation on previously unexposed skin (buttock).	Skin biopsy analysis; mRNA and RT-PCR for matrix metalloprotein-1 (MMP-1) expression 24 h post irradiation.	Sunscreen plus antioxidants protected skin against MMP-1 increase, sunscreen alone did not. No indication of levels of antioxidants, or whether they were able to penetrate into skin layers. Multi-component antioxidant mix.	[153]
In vivo study of 15 healthy adults. Protective effect of vitamin C mixtures (vit. C, vit. E, ferulic acid OR vitamin C, phoretin, ferulic acid) on ozone exposure on forearms.	Skin biopsy analysis; 4-HNE and 8-iso prostaglandin levels, immunofluorescence for NF-kB p65, cyclooxygenase-2, matrix metalloprotein-9 (MMP-9), type III collagen. After 5 days of 0.8 ppm ozone for 3h/d.	Vitamin C mixture reduced ozone induced elevation in lipid peroxidation products, NF-kB p65, cyclooxygenase-2 expression and completely prevented MMP-9 induction by ozone. No indication of levels of antioxidants, or whether they were able to penetrate into skin layers. Multi-component antioxidant mix.	[187]
Test of topical silicone gel with vit. C on scar formation in a population of 80 Asian people. Gel applied for six months after operation.	Scar formation monitored by modified Vancouver Scar Scale (VSS) as well as erythema and melanin indices by spectrophotometer.	Vit. C decreased scar elevation and erythema, decreased melanin index. Improved wound healing (stitch removal).	[166]
